# The effect of micro-osteoperforations on the rate of maxillary incisors' retraction in orthodontic space closure: a randomized controlled clinical trial

**DOI:** 10.1186/s40510-023-00505-z

**Published:** 2024-02-12

**Authors:** Carolina Morsani Mordente, Dauro Douglas Oliveira, Juan Martin Palomo, Polyana Araújo Cardoso, Marina Araújo Leite Assis, Elton Gonçalves Zenóbio, Bernardo Quiroga Souki, Rodrigo Villamarim Soares

**Affiliations:** 1 Graduate Program in Dentistry, School of Dentistry, Pontifical Catholic University of Minas Gerais, Belo Horizonte, MG Brazil; 2 Graduate Program in Dentistry, Department of Orthodontics, Pontifical Catholic University of Minas Gerais, Belo Horizonte, MG Brazil; 3 Department of Orthodontics, School of Dental Medicine, Case Western Reserve University, Cleveland, OH USA; 4 Graduate Program in Dentistry, School of Dentistry, Pontifical Catholic University of Minas Gerais, Avenida Dom José Gaspar, 500, Prédio 46, Sala 101, Belo Horizonte, MG 30535-901 Brazil

**Keywords:** Orthodontic appliances, Tooth movement techniques, Root resorption

## Abstract

**Background:**

This single-centered randomized controlled clinical trial aimed to evaluate the effectiveness of micro-osteoperforations (MOPs) in accelerating the orthodontic retraction of maxillary incisors.

**Methods:**

Forty-two patients aged 16–40 were recruited and randomly assigned into two groups, one which underwent MOPs (MOPG) in the buccal and palatal region of all maxillary incisors immediately before the start of retraction and one which did not (CG). Eligibility criteria included the orthodontic need for maxillary first premolars extraction and space closure in two phases. The primary outcome of the study consisted of measuring the rate of space closure and, consequently, the rate of incisors’ retraction using digital model superimposition 14 days later and monthly thereafter for the next 4 months. The secondary outcomes included measuring anchorage loss, central incisors’ inclination, and root length shortening, analyzed using cone beam computed tomography scans acquired before retraction and 4 months after retraction. Randomization was performed using QuickCalcs software. While clinical blinding was not possible, the image’s examinator was blinded.

**Results:**

Twenty-one patients were randomly assigned to each group. However, due to various reasons, a total of 37 patients (17 male and 20 female) were analyzed (mean age: 24.3 ± 8.1 years in the MOPG; 22.2 ± 4.2 years in the CG) during the trial. No statistically significant difference was found between the MOPG and the CG regarding the incisors’ retraction measured at different time points at the incisal border (14 days, 0.4 mm vs. 0.5 mm; 1 month, 0.79 mm vs. 0.77 mm; 2 months, 1.47 mm vs. 1.41 mm; 3 months, 2.09 mm vs. 1.88 mm; 4 months, 2.62 mm vs. 2.29 mm) and at the cervical level (14 days, 0.28 mm vs. 0.30 mm; 1 month, 0.41 mm vs. 0.32 mm; 2 months, 0.89 mm vs. 0.61 mm; 3 months, 1.36 mm vs. 1.10 mm; 4 months, 1.73 mm vs. 1.39 mm). Similarly, no statistically significant differences were detected in the space closure, anchorage loss, central incisors’ inclination, and radicular length between groups. No adverse effect was observed during the trial.

**Conclusions:**

MOPs did not accelerate the retraction of the maxillary incisors, nor were they associated with greater incisor inclination or root resorption.

*Trial registration* ClinicalTrials.gov NCT03089996. Registered 24 March 2017—https://clinicaltrials.gov/ct2/show/NCT03089996.

## Introduction

Long treatment duration is considered a drawback in orthodontics [1]. The prolonged time needed to achieve planned goals is due to the limited rate of tooth movement, which may range from 0.35 to 2.04 mm/month [2]. This situation often leads patients to refuse undergoing orthodontic treatment [3]. Prolonged treatment time also leads to additional biological risks, such as developing white spot enamel lesions, dental caries [4], and higher chances of root resorption [5, 6]. Moreover, since maintaining patients’ satisfaction and cooperation during long-term treatments may be challenging [7], methods aiming to decrease treatment duration without compromising the outcomes have been an important objective of contemporary orthodontic research [1].

One of the important variables that determine the rate of tooth movement is the patient’s bone metabolism and thus the rate of bone resorption/apposition, which is directly related to both osteoclastic and osteoblastic activity [8, 9]. Therefore, factors recruiting osteoclast precursors from blood circulation and stimulating the differentiation of these cells into mature osteoclasts play a significant role in regulating orthodontic tooth movement [3]. Micro-osteoperforations (MOPs), a minimally invasive surgical technique involving multiple transmucosal perforations within the alveolar bone near the region of the desired tooth movement, has been described as an alternative method of inducing the acceleration of orthodontic treatments [3]. The biological mechanism that may explain MOPs’ potential to accelerate orthodontic tooth movement is related to the increase in cytokine and chemokine expression after the surgical procedure, leading to increased osteoclastic activity and bone remodeling [3]. However, although the facilitation of tooth movement may reduce the risk of apical tooth resorption, the increased alveolar bone turnover and osteoclastic activity have been found to exacerbate the root resorption process, which is an undesirable outcome in orthodontics [10].

Recent systematic reviews analyzing the effects of MOPs have emphasized the low quality of the published studies due to small sample sizes, with a high risk of bias as a possible reason for the controversial results [11, 12]. The lack of standardized measurements and the use of unstable fiduciary landmarks has resulted in inconsistent findings and, consequently, questionable results [11, 12]. In this context, the three-dimensional superposition of digital dental models is an innovative tool to quantify individual dental changes between two or more records [13, 14]. Considering the risk of increased apical root resorption, previous studies using different methods of evaluation, such as radiographs, cone beam computed tomography (CBCT), and microcomputed tomography, present controversial results about the effects of MOPs [15–18].

The scientific evidence on the acceleration of orthodontic retraction of maxillary incisors following MOPs is limited and controversial. The development of a randomized controlled clinical trial (RCCT) to investigate this topic is needed since the only previous RCCT that evaluated maxillary incisor retraction used another technique (piezocision-assisted flapless corticotomy) [19]. That study demonstrated the effectiveness of piezocision corticotomy in its ability to accelerate the retraction of four maxillary incisors, reduce the treatment duration, maintain anchorage, and enhance root torque control [19]. Therefore, the current RCCT brings new information by investigating the effectiveness of MOPs in accelerating the retraction of maxillary incisors in orthodontic treatments that require the extraction of maxillary first premolars.

### Specific objectives or hypotheses

The primary purpose of this study was to assess the impact of MOPs on the maxillary incisor retraction rate and space closure rate over a 4-month period. The secondary purpose was to evaluate the incisors’ inclination, as well as the occurrence of root resorption and anchorage loss. The null hypothesis was that MOPs do not accelerate maxillary incisor retraction compared with conventional orthodontic methods.

## Materials and methods

This clinical trial was written according to the CONSORT (Consolidated Standards of Reporting Trials) guidelines for the improvement of reporting quality.

### Trial design

This was a randomized (allocation rate 1:1) and controlled clinical trial. During the analysis of the CBCT scans to plan the MOP sites, the interradicular spaces in the maxillary anterior region were found to be small in two patients. Thus, the placement of these two individuals in the comparison group (CG) was conducted non-randomly due to the increased probability of injuring the root surfaces. This trial was registered on ClinicalTrials.gov.

### Participants and study setting

Ethical approval was obtained from the institutional review board of the Pontifical Catholic University of Minas Gerais. Participants were recruited by screening patients seeking orthodontic treatment at the Graduate Program in Orthodontics at the Pontifical Catholic University of Minas Gerais.

Inclusion criteria were: (1) male and female subjects, (2) older than 16 years of age, (3) indication of orthodontic maxillary incisor retraction, and (4) presence of all permanent maxillary teeth (except for the third molars). Exclusion criteria were: (1) diseases and/or use of medications that could affect bone biology, (2) pregnancy, (3) poor oral hygiene, (4) previous orthodontic treatment, (5) evidence of bone loss, (6) active periodontitis, (7) smoking, (8) presence of syndromes or cleft palate, (9) severe crowding, (10) severe Class II malocclusion (ANB > 7º, overjet > 10 mm), (11) hyperdivergency (SNGoGn > 38º). After the purpose of the intervention and the associated risks and potential benefits were explained, eligible patients who agreed to take part in the study signed an informed consent form.

### Interventions

#### Orthodontic procedures

Initially, subjects were referred to the periodontics clinic to evaluate their periodontal conditions and receive regular oral hygiene care. After extraction of the first maxillary premolars, orthodontic treatment started with fixed appliances in both the maxillary and mandibular arches (0.022-in, Edgewise, American Orthodontics, Sheboygan, WI, USA). After alignment and leveling, interradicular temporary anchorage devices (TADs) (1.5 mm width, 6 mm length, Morelli, Sorocaba, Brazil) were installed at the buccal mucogingival margin between the maxillary second premolars and first molars. TADs were used as direct anchorage to prevent anchorage loss of the posterior teeth during the retraction of the canines and incisors.

**Fig. 1:**
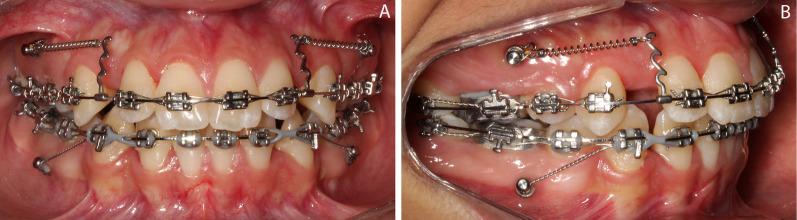
Maxillary incisors retraction mechanics. **A** Frontal view; **B** lateral view

The retraction of the maxillary incisors was begun after a bilateral Class I canine relationship was achieved. Adequate overbite and overjet were also required to avoid anterior occlusal trauma during incisor retraction. A continuous 0.017 × 0.025″ stainless steel wire was used, and 10-mm-high crimpable hooks were installed distally to the maxillary lateral incisors, allowing the application of the retraction force as close as possible to the center of resistance of the four maxillary incisors. Closed nickel-titanium (Ni–Ti) springs (Sentalloy 200 g, Dentsply/GAC, York, PA, USA) with 200 g of force were connected from the hooks to the TADs bilaterally (Fig. 1).

Patients returned 2 weeks after the start of incisor retraction and monthly thereafter during a 4-month period. In all clinical appointments, the force applied was recorded with a high-precision dynamometer (Correx, Haag-Streit AG, Koeniz, Switzerland), and the NiTi springs were adjusted when necessary to maintain 200 g of retraction force. The installation and adjustments of orthodontic mechanics in all subjects were performed by a single, experienced, and trained orthodontist (C.M.M.).

#### Micro-osteoperforations

**Fig. 2:**
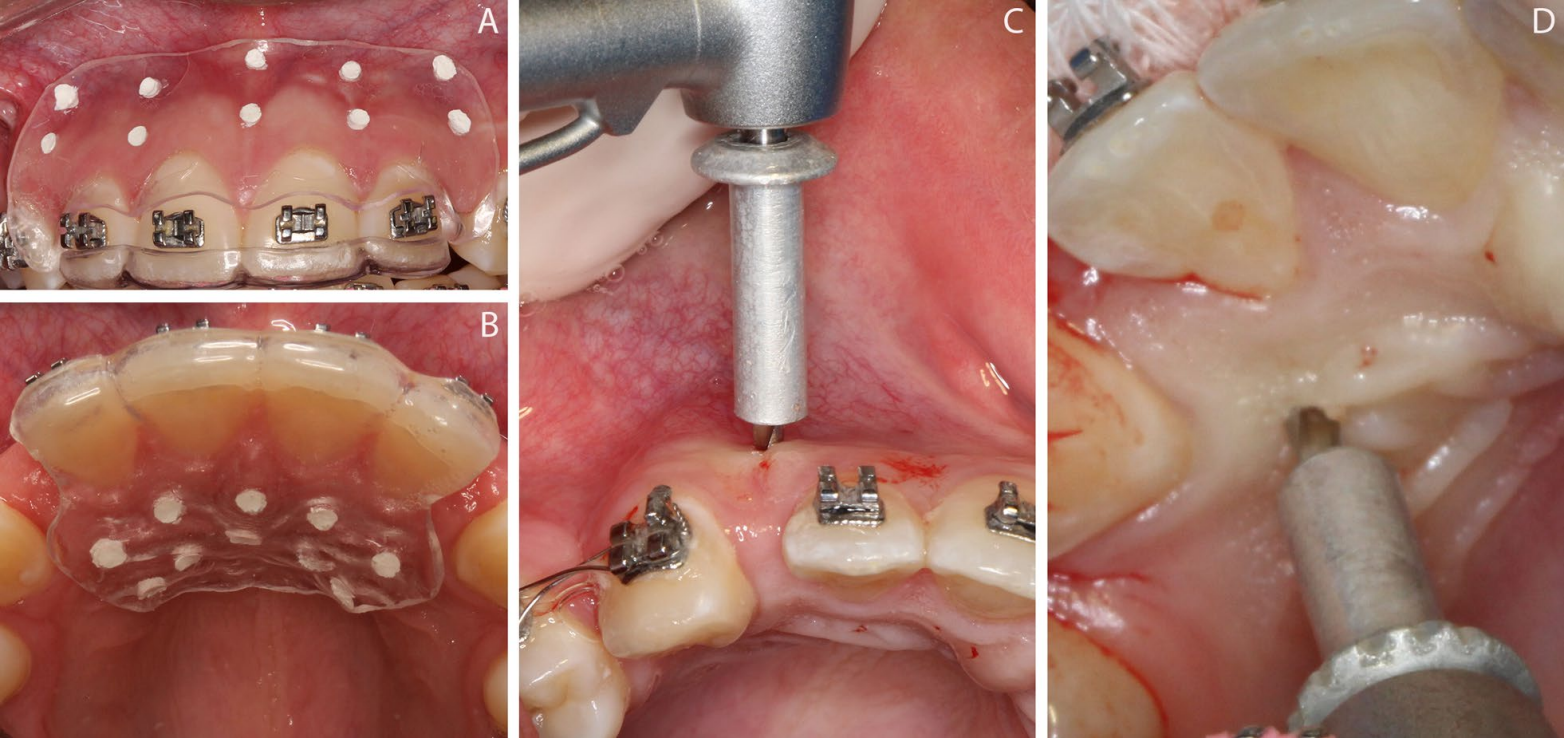
Micro-osteoperforations (MOPs). **A** and **B** Surgical guides; **C** and **D** MOPs on the buccal and palatal surfaces using a cylindrical surgical drill, to which the cursor was added to limit the depth of the perforation, 3 mm on the buccal surface (**C**) and 5 mm on the palatal surface (**D**)

All MOPs were performed only once and on the same day that the maxillary incisors’ retraction was begun. Patients were instructed to rinse with 0.12% chlorhexidine for 1 min before the procedure. Local anesthesia and MOPs were carried out by a single, experienced, and trained periodontist (E.G.Z), following the same procedure:

1. Individualized surgical guides made with a 1-mm Essix ACE thermoformed plastic (Essix, Dentsply Sirona, Charlotte, NC, USA) were used during MOPs performance, based on the initial CBCT images (Fig. 2A, B).
2. MOPs were performed with a 1.6-mm diameter stainless steel surgical bur, perpendicular to the alveolar bone, 3 mm deep on the buccal surface [20] and 5 mm deep on the palatal region, due to the greater thickness of the soft tissue in this region. The perforation depth was controlled and standardized by a cursor developed and patented by this research group (Fig. 2C, D).
3. Nine MPOs were performed on both the buccal and palatal regions. Two MOPs were aligned vertically, distally from each maxillary incisor. Due to the proximity of the roots in the cervical third, only the most apical perforation was performed between the two central incisors. The first MOP was performed 6 mm away from the gingival margin, and the second was performed 5 mm from the first [20].

At the end of the procedure, patients were instructed about postoperative care regarding the maintenance of good oral hygiene [20] and directed to use 500 mg paracetamol (every 6 h for 2 days) in case of pain.

### Outcomes

Digital models were obtained in “.stl” format (stereolithography) by intraoral scanning (3Shape, Copenhagen, Denmark) after retraction of the canines, immediately before implementing the incisors’ retraction mechanics (T0), 14 days after the beginning of retraction (T1), and 1 (T2), 2 (T3), 3 (T4) and 4 months (T5) after the beginning of retraction. CBCT scans were obtained before the beginning of retraction (T0) and 4 months after (T4), thus preventing any root damage during MOPs. An i-CAT® scanner (Imaging Sciences International, Hatfield, PA, USA) was used with an extended skull FOV (field of view), set within the parameters of 23 cm × 17 cm, 0.3 mm^3^ voxels, 36.90 mA, 120 kV, and an exposure time of 40 s, to generate DICOM (Digital Imaging and Communications in Medicine) files. All measurements were performed by the same orthodontist (C.M.M.), who received specific training from a senior researcher with extensive 3D image analysis experience (B.Q.S.).

### Primary outcome

Identifying the monthly space closure rate was the current trial’s major objective. As space closure is a consequence of the orthodontic retraction of maxillary incisors, the measurement of the anteroposterior displacement of these teeth was considered the primary outcome. The digital models superimposed in the initial CBCT scans provided the reference plans.

**Fig. 3:**
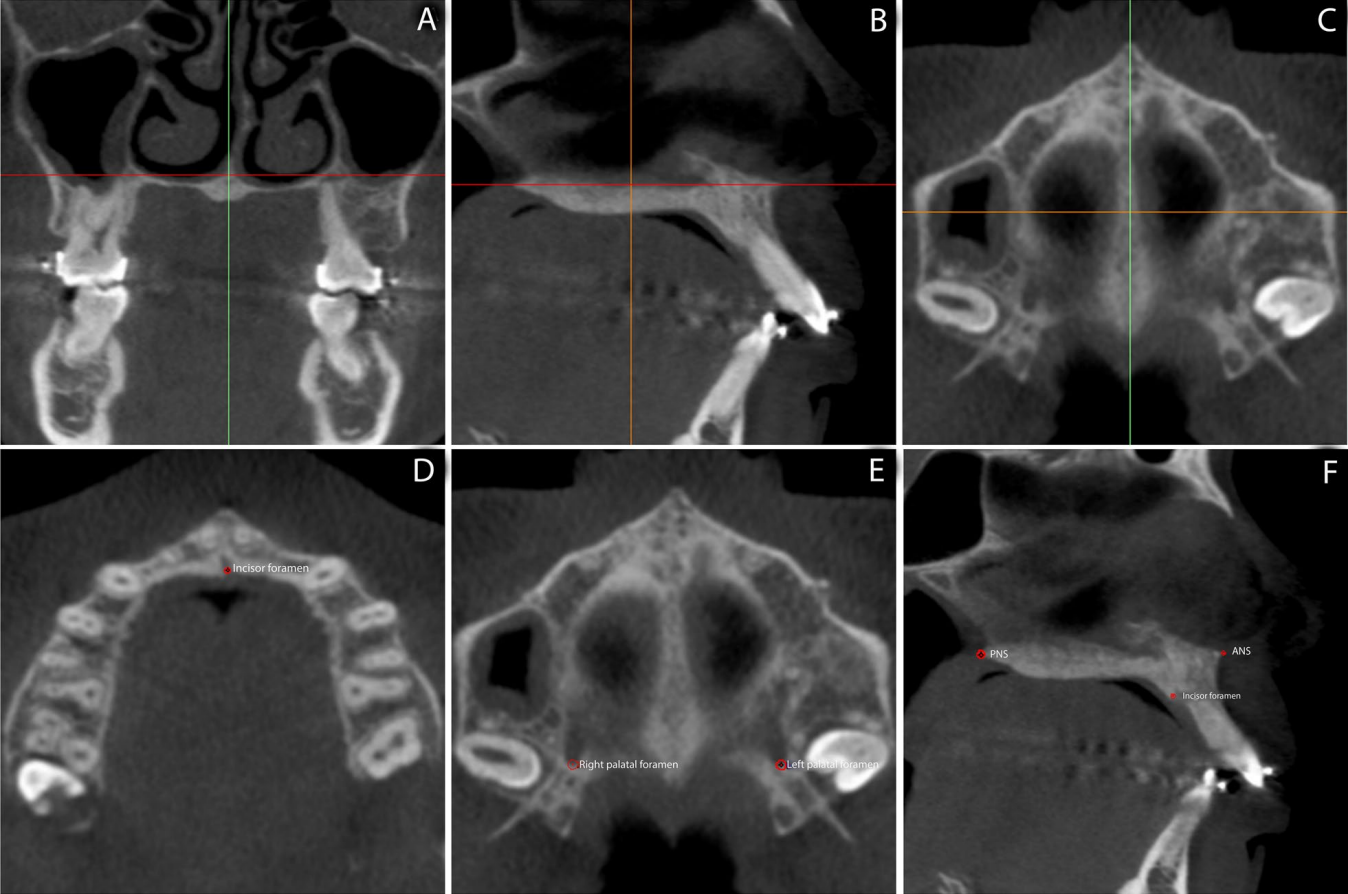
Maxillary orientation. **A**, **B**, and **C** Orientation of the maxilla. **A** Frontal view: zygomatic maxillary sutures oriented parallel to the ground; **B** lateral view: palatal plane oriented parallel to the ground; **C** superior view: ANS and PNS oriented perpendicular to the ground. **D**, **E**, and **F**, landmarks selection to generate the reference planes: incisor foramen (**D**), greater palatine foramen, right and left (**E**), ANS and PNS (**F**)

Using VistaDent 3D software (Dentsply GAC, York, PA, USA), the baseline CBCT scans were oriented as follows: (1) frontal view: line that passes through the right and left zygomatic maxillary sutures selected in the coronal slice passing through the center of the crown of the maxillary first right molar, parallel to the ground (Fig. 3A); (2) lateral view: line passing through the anterior nasal spine (ANS) and posterior nasal spine (PNS) in the mid-sagittal slice, parallel to the ground (Fig. 3B), with the PNS point selected in the same sagittal slice in which the ANS was identified; (3) superior view: line passing through the ANS and PNS points in the axial section, perpendicular to the ground (Fig. 3C).

Using Ortho Analyzer 2019 (3Shape, Copenhagen, Denmark), models were superimposed at T1 through T5 on the oriented baseline model (T0) using the “3D surface-to-surface matching” method and the “three points and one surface” tool, using the third palatal rugae as a reference, as previously described [21]. The maxillary rugae superimposition is considered a reliable reference for the superimposition of digital models [22–24]. Two medial points on the third rugae, bilaterally, and a third point in the middle of the right third rugae were used. The selected surface included the medial region of the third right and left rugae and a posterior expansion over the palatal raphe, with approximately 10 clicks. This region is considered a stable structure even after orthodontic treatment and extraction of premolars [23, 24]. The six maxillary models of each patient were exported from the Ortho Analyzer software after superimpositions.

The digital models were merged into the initial CBCT scans using VistaDent 3D. Initially, models T1–T5 were hidden to allow the superimposition of the T0 model to the CBCT scans. Only the contour of the reference model (T0) was visually active, allowing for the superimposition on the initial CBCT. Then, models T1–T5 were registered on the maxillary rugae of the T0 model to standardize maxillary orientation.

Subsequently, the following reference planes were created using points selected in the initial CBCT scan (Fig. 3D–F): (1) axial plane: formed by the most posterior point of the incisor foramen, selected in the first axial slice in which the foramen is entirely closed, and by the most posterior points of the right and left greater palatal foramen, selected in the first axial section in which the right foramen is closed in the anterior region; (2) sagittal plane: formed by the ANS and PNS points, selected as described above, perpendicular to the axial plane; (3) coronal plane: formed by the same points in the right and left palatal foramen and perpendicular to the sagittal plane.

**Fig. 4:**
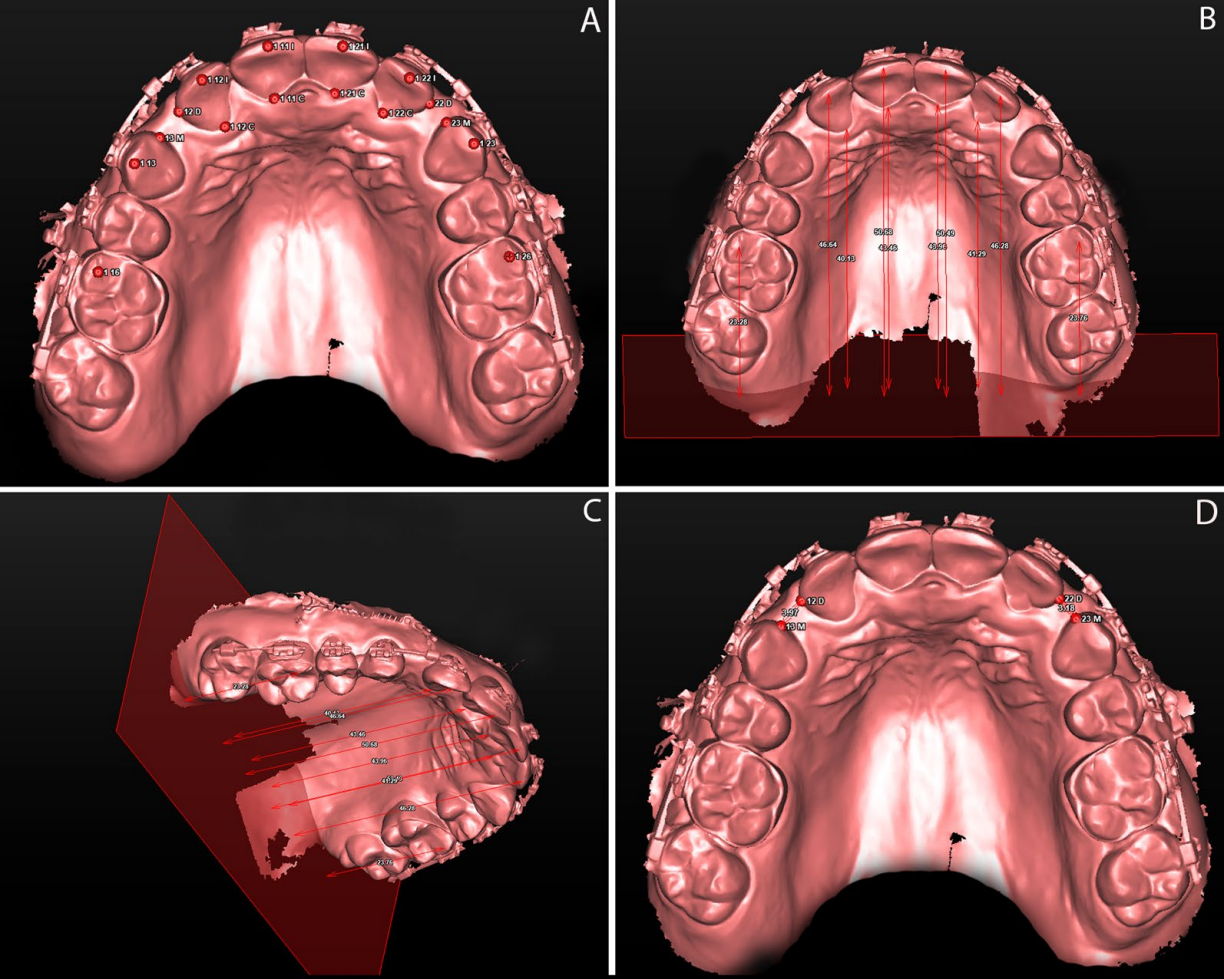
Digital models analysis using VistaDent 3D software. **A** Landmarks selection; **B**, **C**, and **D** measurements

Landmarks were selected in models T0 to T5 (Fig. 4A and Table 1). The anteroposterior incisal displacement was represented by the distance between the midpoint of the incisal edge of the lateral and central incisors (UL2I, UR2I, UL1I, UR1I) to the coronal plane. The anteroposterior cervical displacement was represented by the distance between the lateral and central incisors' midpoint of the cervical palatal margin (UL2C, UR2C, UL1C, UR1C) to the coronal plane (Fig. 4B, C). The maxillary incisors’ displacement is represented by the differences between the measurements obtained in each model (T1–T5) relative to the initial reference model (T0). Since the four maxillary incisors were retracted simultaneously, a single measure was calculated for each patient at each measurement point (T1–T5), indicating the average displacement of the four incisors.

**Table 1:** Definition of landmarks

Landmarks	Definition
UL6	Mesiobuccal cusp of maxillary left first molar
UL2I	Midpoint of the incisal edge of maxillary left lateral incisor
UL2C	Midpoint of the cervical palatal margin of maxillary left lateral incisor
UL1I	Midpoint of the incisal edge of maxillary left central incisor
UL1C	Midpoint of the cervical palatal margin of maxillary left central incisor
UR1I	Midpoint of the incisal edge of maxillary right central incisor
UR1C	Midpoint of the palatal cervical margin of maxillary right central incisor
UR2I	Midpoint of the incisal edge of maxillary right lateral incisor
UR2C	Midpoint of the cervical palatal margin of maxillary right lateral incisor
UR6	Mesiobuccal cusp of maxillary right first molar
UL2D	Distal surface of maxillary left lateral incisor
UR2D	Distal surface of maxillary right lateral incisor
UL3M	Mesial surface of maxillary left canine
UR3M	Mesial surface of maxillary right canine

The distance from the distal surface of the lateral incisors (UR2D and RL2D) to the mesial surface of the canines (UR3M and UL3M) was calculated in both sides to demonstrate space closure with the retraction of the maxillary incisors (Fig. 4D).

#### Secondary outcomes

The secondary outcomes of the study were to measure the mesial movement (anchorage loss) of the maxillary first molars, changes in the buccolingual inclination of the central incisors, and root length changes in the maxillary central incisors.

Anchorage loss was calculated using the distance between the mesiobuccal cusp of the maxillary first molars (UR6 and UL6) and the coronal plane. This figure is represented by the differences between the measurements obtained in each model (T1–T5) relative to the baseline reference model (T0) (Fig. 4B, C).

The changes in the buccolingual inclination and radicular length of the maxillary central incisors were calculated using the CBCTs in the Dolphin Imaging software (Chatsworth, CA, USA). The head orientation of the baseline CBCT was performed as previously described [25]. Then, the final CBCTs were superimposed in the baseline scan using the “voxel-based method,” with the cranial base as reference [26].

**Fig. 5:**
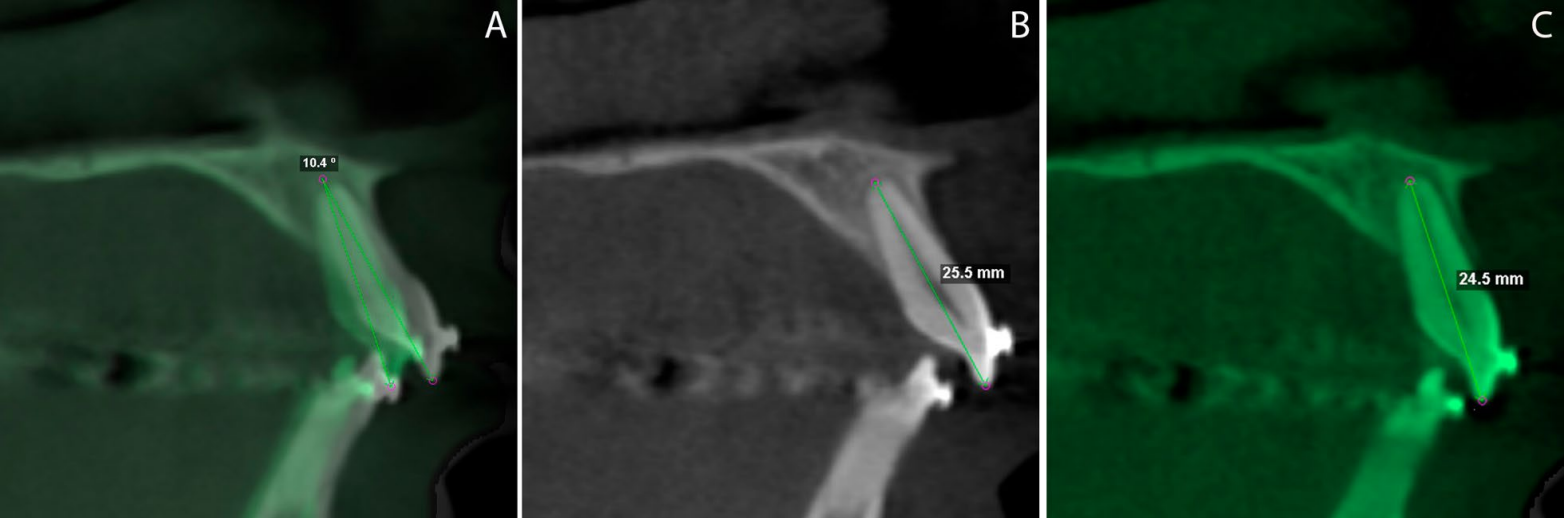
Inclination and changes in length of the central incisors after maxillary retraction. **A** Angle formed by the long axis of the central incisors at T0 and after 4 months; **B** baseline; **C** after 4 months

The buccolingual inclination of the maxillary central incisors was then assessed using the angle formed by the long axis of each central incisor in the baseline and final CBCTs. The long axis of the incisors was determined in the sagittal view, in which the most extended length of these teeth was seen (Fig. 5A).

The initial and final lengths of the central incisors were measured by taking the distance from the incisal border and root apex as references, allowing the calculation of dimensional shortening of the roots and thus the occurrence of root resorption (Fig. 5B, C).

### Sample size calculation

Based on a previous study [27], 0.21 mm was used as the standard deviation of the monthly rate of orthodontic space closure (primary outcome) to calculate the sample size. The study adopted a 1:1 ratio between groups, an effect size of 1, alpha of 5%, and power of 80%. Using the G*Power software (version 3.1, Heinrich-Heine-Universität Düsseldorf, Düsseldorf, Germany), these parameters thus called for a sample of 34 individuals (17 in the CG and 17 in the MOPG). Four individuals were added to each group to compensate for possible losses. Therefore, the research sample size was composed of 42 individuals (21 in each group).

### Randomization

Randomization was performed by generating a random sequence using QuickCalcs (GraphPad Software, Inc., La Jolla, CA, USA). Since the performance of MOPs required prior planning using tomographic exams and the preparation of surgical guides, the periodontist was informed of the participants’ allocation 1 week before the procedure. Concealing this allocation from the orthodontic team prevented selection bias and protected the assignment sequence until allocation.

### Blinding

Blinding of patients and orthodontists was not possible during the period of evaluation of the maxillary incisors’ retraction. However, blinding was ensured at the measurement stage (data collection), as the investigator (C.M.M.) was blinded to where MOPs were applied by coding all digital models.

### Statistical analysis

The student t test and chi-square test were used for intergroup comparisons of age and sex distribution, respectively. All measurements were repeated in 60 digital models and in 20 CBCT scans that were randomly selected after 2 weeks. The intraclass correlation coefficient (ICC) ranged from 0.86 to 0.99, indicating good intra-examiner reproducibility. The systematic error was calculated using the paired t test. No significant results were found. The D'Agostino-Pearson test demonstrated that the data obtained presented a normal distribution. Means and standard deviations were then calculated for all variables. The intra- and intergroup comparisons of the displacement of the incisors and molars and the space closure during the time period were performed using the two-way ANOVA test, followed by Bonferroni's post hoc test. Intergroup comparisons of the central incisors’ changes in inclination and length were performed using the *t* test. The paired *t* test was used for intragroup evaluation of the changes in central incisor length. The analyses were processed using Graph Pad Prism 5.0 (GraphPad Software, San Diego, CA, USA) and a significance level of 5%.

## Results

### Recruitment and participant flow

**Fig. 6:**
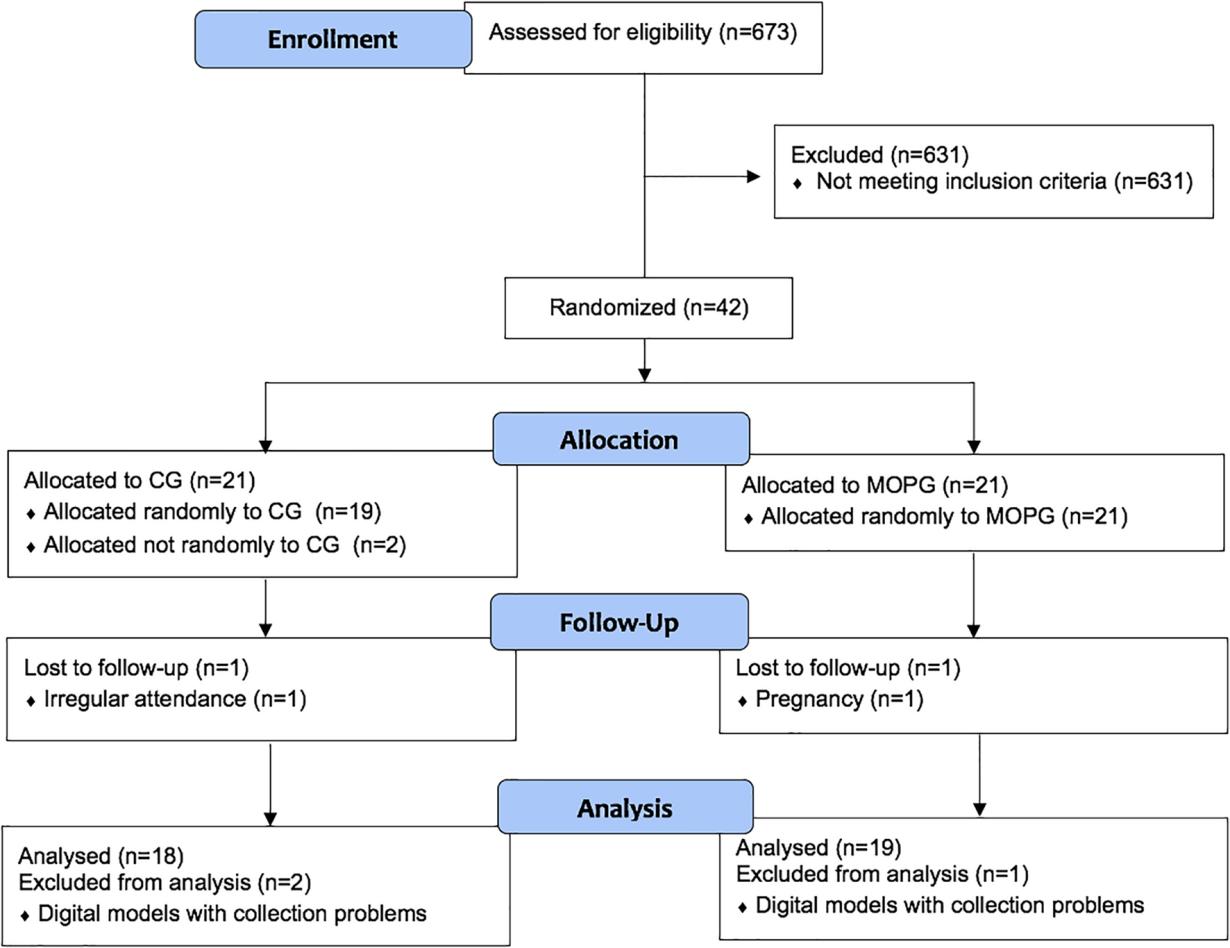
CONSORT flowchart

Patients were recruited from February 2016 to June 2017, with the final data collection performed in December 2019. A total of 42 patients were randomly assigned with a 1:1 allocation ratio and received the treatment corresponding to their group. However, one subject from each group was excluded in the course of the trial due to irregular attendance and due to pregnancy. In addition, during the data analysis, a subset of scans was rendered unusable due to equipment failures, resulting in the inability to retrieve the corrupted data and in the exclusion of three subjects from the sample (two in the CG and one in the MOPG). Therefore, this clinical trial was conducted with 18 subjects in the CG and 19 in the MOPG. The participant flow during the trial is described in the CONSORT flowchart (Fig. 6).

### Baseline data

Data regarding the age, sex, and type of malocclusion in each group are described in Table 2. The mean age was 22.2 ± 4.2 years in the CG and 24.3 ± 8.1 years in the MOPG. Analysis of the age and sex distribution between groups did not reveal statistically significant differences.

**Table 2:** Baseline characteristics

	CG	MOPG	*P* value
Participants	18	19	
Age (mean and SD)	22.2 (4.2)	24.3 (8.1)	ns*
Sex			
Male	9	8	ns**
Female	9	11	
Malocclusion			
Class I	6	8	
Class II division 1	6	5	
Class II division 2	6	6	
Anterior crowding	7	11	
Deep bite	7	5	
Open bite	4	1	
Biprotrusion	3	7	

^*^ T test P value; ** Chi-square test P Value; CG, comparison group; MOPG, micro-osteoperforations group; SD, standard deviation; M, male; F, female; ns, non-significant (P ≥ 0.05)

### Outcomes analysis

All data referring to a total of 37 individuals were analyzed. Therefore, a total of 222 models and 75 CBCTs were analyzed. During the evaluation period, there was no loss of TADs or occlusal trauma due to the retraction movement.

#### Primary outcome

In the CG, the mean values of incisor displacement at the incisal level at T1, T2, T3, T4, and T5 were 0.50 mm, 0.77 mm, 1.41 mm, 1.88 mm, and 2.29 mm, respectively. Intragroup analysis revealed that significant differences were only found between T2 and T3.

In the MPOG, the mean values found for the incisor displacement at the incisal level at the same points were 0.40 mm, 0.79 mm, 1.47 mm, 2.09 mm, and 2.62 mm, respectively. Intragroup analysis revealed statistically significant differences between T2 and T3 and between T3 and T4.

In the CG, the mean values of incisor displacement at the cervical level at the same evaluation points were 0.30 mm, 0.32 mm, 0.61 mm, 1.10 mm, and 1.39 mm, respectively. Intragroup analysis revealed that statistically significant differences were only found between T3 and T4.

In the MPOG, the mean values of incisor displacement at the cervical level at the same points were 0.28 mm, 0.41 mm, 0.89 mm, 1.36 mm, and 1.73 mm, respectively. Intragroup analysis revealed statistically significant differences between T1 and T2, between T2 and T3, and between T3 and T4.

Intergroup analysis at each evaluation point (T1–T5) did not show statistically significant differences between the CG and the MPOG in the displacement of the incisors and space closure (Table 3).

**Table 3:** Teeth displacement and space closure

Groups	AP displacement	2 weeks (T0-T1)	1 month (T0-T2)	2 months (T0-T3)	3 months (T0-T4)	4 months (T0-T5)	*P value
CG	Incisors—incisal	0.50 ± 0.60	0.77 ± 0.67	1.41 ± 0.84	1.88 ± 0.74	2.29 ± 1.09	ns ^a,c,d^ < 0.05 ^b^
	Incisors—cervical	0.30 ± 0.59	0.32 ± 0.21	0.61 ± 0.27	1.10 ± 0.38	1.39 ± 0.61	ns ^a,b,d^ < 0.05 ^c^
	Space closure (R)	0.16 ± 0.31	0.29 ± 0.29	0.68 ± 0.56	1.04 ± 0.74	1.32 ± 1.02	ns
	Space closure (L)	0.30 ± 0.44	0.41 ± 0.47	0.75 ± 0.60	1.14 ± 0.79	1.49 ± 1.01	ns
	UR6	0.14 ± 0.60	0.05 ± 0.32	0.04 ± 0.29	− 0.02 ± 0.36	− 0.16 ± 0.69	ns
	UL6	0.17 ± 0.73	0.02 ± 0.31	0.01 ± 0.38	− 0.04 ± 0.32	− 0.06 ± 0.49	ns
MOPG	Incisors—incisal	0.40 ± 0.27	0.79 ± 0.37	1.47 ± 0.63	2.09 ± 0.68	2.62 ± 0.75	ns ^a,d^ < 0.05 ^b,c^
	Incisors—cervical	0.28 ± 0.24	0.41 ± 0.27	0.89 ± 0.40	1.36 ± 0.43	1.73 ± 0.38	ns ^a^ < 0.05 ^b,c,d^
	Space closure (R)	0.20 ± 0.34	0.46 ± 0.50	0.84 ± 0.80	1.33 ± 0.98	1.70 ± 1.20	ns
	Space closure (L)	0.34 ± 0.35	0.60 ± 0.43	1.08 ± 0.70	1.60 ± 0.84	2.01 ± 0.98	ns
	UR6	− 0.14 ± 0.71	0.08 ± 0.21	0.13 ± 0.32	0.12 ± 0.30	0.04 ± 0.43	ns
	UL6	0.01 ± 0.35	0.13 ± 0.30	0.10 ± 0.43	0.14 ± 0.22	0.08 ± 0.33	ns
		ns	Ns	ns	ns	ns	*P value

Values in millimeters. *Two-way ANOVA’s test *P* value (intragroups’ comparisons; intergroups’ comparisons), *AP* anteroposterior; *CG*, comparison group; MOPG, micro-osteoperforations group; R, right; L, left; ns, non-significant (P ≥ 0.05); ^a^ 2 weeks *versus* 1 month; ^b^ 1 month *versus* 2 months; ^c^ 2 months *versus* 3 months; ^d^ 3 months *versus* 4 months

#### Secondary outcome

The intra- and intergroup analysis of anchorage loss did not show significant differences in any of the evaluation periods (Table 3). Similarly, there were no statistically significant differences between the MOPG and the CG regarding the inclination of the maxillary central incisors (Table 4). Although the intragroup analysis showed a significant decrease in the length of the central incisors in both groups, the intergroup comparison did not reveal significant differences between the MOPG and the CG (Table 5).

**Table 4:** Central incisors’ angulation

Central incisors	CG	MPOG	*P* value*
UR1	5.66 ± 4.70	6.86 ± 3.84	ns
UL1	5.29 ± 4.60	6.07 ± 3.60	ns

Values in degrees. *T test *P* value; ns, non-significant (*P* ≥ 0.05)

**Table 5:** Incisors’ length

Groups	Central incisors	Initial	Final	Mean of differences (Final—Initial)	*P* value*
CG	UR1	23.87 ± 2.19	23.12 ± 2.22	− 0.77 ± 0.38	< 0.05
	UL1	23.77 ± 2.05	23.01 ± 1.92	− 0.76 ± 0.54	< 0.05
MOPG	UR1	23.16 ± 1.14	22.29 ± 1.32	− 0.87 ± 0.48	< 0.05
	UL1	22.76 ± 1.57	21.98 ± 1.99	− 0.78 ± 0.93	< 0.05
				ns	*P* value**

Values in millimeters. *Paired *t* test *p* value, intragroups comparison; **Independent *T* test *P* value, intergroups comparison; ns, non-significant (*P* ≥ 0.05)

#### Harms

Participants did not report any adverse effects related to the MOPs surgical procedure.

## Discussion

In recent years, MOPs have caught the attention of clinicians and researchers because it is a minimally invasive procedure that may accelerate tooth movement [28]. However, the current scientific evidence about the effects of MOPs is limited, and there is no conclusive research regarding the real benefit of this procedure in accelerating orthodontic tooth movement [3, 11, 27]. Therefore, this RCCT was designed to contribute to the scientific evidence regarding the effects of MOPs on the retraction of maxillary incisors. Our findings were contrary to those reported by the only previous RCCT to look into the acceleration of maxillary incisor retraction, although that study examined a different minimally invasive technique called piezocision-assisted flapless corticotomy [19]. While this previous study showed a major improvement in orthodontic outcomes by using piezocision corticotomy, our data regarding the use of MOPs yielded different results.

### Primary results

In both the MOPG and the CG, significant retraction of the incisors was achieved with orthodontic forces. However, although the incisors’ anteroposterior displacement was slightly higher in the MPOG compared to the CG, these differences were not statistically or clinically significant (T1–T5) and did not lead to faster incisor retraction. Regarding the space closure on both sides, intra- and intergroup comparisons did not show significant differences, indicating no benefit from MOPs in terms of accelerating the retraction of the maxillary incisors.

Comparing the current findings with previous studies is difficult, since the literature contains no investigations of the effects of MOPs on the retraction of maxillary incisors. Thus, we can only contrast our data with studies evaluating the effects of MOPs on the orthodontic movement of canines and en-masse retraction of the six anterior teeth.

In this context, one study reported a 2.3-fold increase in the canine distalization movement after performing three MOPs distal to the canine using Propel®, an instrument designed specifically for this procedure [3]. However, that study presents a significant risk of bias due to its small sample size (*n* = 10), short follow-up period (28 days), and a lack of information regarding randomization. In addition, using the lateral incisor as a reference to measure the canine's movement is unreliable since it does not represent a stable structure and can move during canine retraction.

In previous studies wherein MOPs were repeated every 28 days, a significant increase in the rate of orthodontic tooth movement was reported [27, 29]. However, when the measurement methods of these studies are analyzed, some limitations can be observed. One study evaluated the en masse retraction of the anterior teeth by measuring the extraction space closure instead of the teeth’s displacement [27]. Since skeletal anchorage was not used, this approach entails an important bias related to the anchorage loss’s contribution to the anterior space closure [27]. Another investigation analyzed the displacement of distalized canines with a digital caliper placed directly in the patient’s mouth, using TADs as a reference [29]. Notably, this method does not allow measurements to be performed with accuracy and good reproducibility and does not allow blinding during data collection. Moreover, since TADs were used in orthodontic mechanics, they do not represent reliable reference structures for measuring tooth movement. In the same investigation, only minor differences in tooth movement were found when MOPs were performed at intervals of 4, 8, and 12 weeks, indicating that repeated MOPs play an insignificant role in the acceleration of tooth movement [29].

Two previous studies that used a single-step MOPs application before canine distalization reported a significant increase in the rate of tooth movement in the first 30 days [30] and 45 days [31]. Thereafter, no difference was found after 3 months [31] and 4 months [30]. In another investigation in which the retraction of the six anterior teeth was associated with MOPs performance distally and mesially to the canines, significant acceleration was only found in the first month, with no difference in the second to the fourth month [28]. Similar to our results, an investigation that used single-step MOPs application before the space closure of extracted premolars found that although tooth displacement was slightly higher in the experimental group, this result was not statistically or clinically relevant [32]. On the other hand, another study that repeated MOPs monthly until the space closure did not show any change in the rate of full space closure during en-masse retraction reported a negative impact on the participants’ quality of life [33]. These findings call into question the clinical benefit of MOPs in terms of the amount and duration of acceleration over the entire treatment period and the possible impact on patients’ quality of life.

The results of the present study corroborate the findings of other studies that used the superimposition method of digital models to evaluate MOPs’ effects on tooth movement and that similarly reported the absence of significant acceleration [20, 34]. So far, only one investigation has considered anteroposterior movement of canines alone, using the coronal plane as a reference, as in the present study [34]. When 3D Euclidean measurements are taken, the tooth displacement in the three spatial planes is considered. Therefore, higher values can give a false positive regarding the acceleration of tooth movement, since some components of this displacement might not be in the direction intended by the applied force, for instance due to side effect extrusion.

While some previous studies only performed perforations on the buccal surface [3, 29, 34], one study compared the rate of canine distalization of buccal-only MOPs and that of MOPs conducted on both the buccal and palatal surface [35]. This latter study reported a significant increase in the rate of canine retraction in the group that underwent the procedure on both surfaces. The authors attributed this finding to the additional surgical trauma stimulating a higher expression of inflammatory markers and osteoclast activity. However, in the present study, even with a greater number of perforations (18) on both surfaces, a significant increase in the rate of incisor retraction was not found. The stimulation of the palatal region’s cortical bone was based on the fact that this tissue needs to be reabsorbed for incisors’ retraction movement. However, the maintenance of the periosteum seems to contribute to the ineffectiveness of minimally invasive procedures in accelerating tooth movement [36, 37]. In this regard, it has been demonstrated that opening a surgical flap plays an important role for activating the rapid acceleratory phenomenon (RAP) since the displacement of the periosteum influences the blood supply of the cortical bone [38]. These ischemic conditions, associated with trauma, result in osteoblasts’ death on the bone surface. Due to lack of nutrition, the cortical repair capacity is compromised, and the cells of the bone marrow are recruited, elevating the degree of tooth movement [36]. This theory is supported by the results of a previous study that observed no acceleration of tooth movement for 7-mm-deep perforations in comparison to 4-mm-deep perforations, suggesting that increasing the surgical trauma by deepening MOPs is not more effective for the acceleration of tooth movement [39].

### Secondary results

Evaluating the inclination of the incisors with the retraction movement is critical since lingual inclination can give a false impression of acceleration of the orthodontic movement. The results of the present study revealed that MOPs do not significantly influence the inclination of the central incisors during the retraction mechanics. This result contrasts with the one previous study that used CBCT to conduct this assessment, describing more bodily movement and less inclination relative to the control group, although the acceleration of canine distalization did not occur [34].

Alveolar corticotomy has been suggested to decrease the incidence of root resorption [40], an undesirable and unpredictable orthodontically induced effect [10]. However, there is limited scientific evidence in the literature to prove the association between minimally invasive surgical procedures to accelerate tooth movement and the occurrence of root resorption [12]. The results of the present study revealed that in both groups, the decrease in the length of the central incisors was statistically significant. Nevertheless, in line with previous studies that measured the root length of distalized canines [16, 20, 34] and of the six anterior teeth after the extraction of the premolars, with or without MOPs [15], the present study’s intergroup comparison did not reveal significant root resorption. Recently, Chandorikar and Bhadi [18] presented an RCCT assessing the impact of MOPs on orthodontically induced inflammatory root resorption during en-masse retraction of maxillary and mandibular anterior teeth. They did not find an increased risk of root resorption when the orthodontic movements were combined with a single application of MOPs. Elkalza et al. [41] evaluated the length of canines after orthodontic retraction, comparing the effect of MOPs and piezocision. In that study, it was found that the piezocision group presented more significant root damage.

Significant root resorption has been reported in studies employing microcomputed tomography to analyze volumetric root loss in extracted premolars after applying either MOPs [17] or piezocision [42]. The observed outcome can be attributed to microtomography’s outstanding accuracy. Furthermore, previous studies have focused on detecting resorption craters across the entire radicular surface [17, 42]. In contrast, the present study specifically investigated the total length of the anterior teeth, which implies that only the measurement of apical resorption was conducted.

In order to minimize anchorage loss during the retraction of the maxillary incisors, TADs were installed in the mesial region of the maxillary first molars, due to good space availability and bone quality [43]. The anchorage loss observed in the present study was not clinically relevant and was within the values described in the literature, ranging from 0.06 to 0.78 mm when TADs were used [44]. The intergroup comparisons of anchorage loss between the MOPG and the CG revealed no significant differences between groups, suggesting that the skeletal anchorage minimized molar movement.

### Limitations

A split-mouth design was not possible in the current trial because it conducted simultaneous retraction of the four maxillary incisors. Therefore, individuals’ biological variability may be a factor. To counter this limitation, a robust and homogeneous sample was adopted. In addition, the distribution of the participants was similar in age and sex, and the orthodontic treatment was conducted by the same orthodontist.

The relocation of the two participants from the MOPG to the CG can be viewed as a limitation in the context of this research. Nevertheless, this change was necessary to prevent any harm to the root of the incisors. The value of reporting this issue lies in the fact that while any minimally invasive approach may attract the interest of health professionals due to its conservative philosophical basis, risks are always present, and MOP sites must be carefully planned and not overlooked.

### Generalization

The results of the present study suggest that MOPs do not induce acceleration of the orthodontic movement of the maxillary anterior teeth during retraction. Moreover, MOPs do not interfere in the incisors’ inclination, in the development of root resorption, or in anchorage loss. Therefore, the present study suggests a lack of benefit of using this surgical procedure when planning the retraction of maxillary incisors. However, these results cannot be generalized since the evaluation was conducted in a single center.

## Conclusion

- MOPs performed in a single step did not accelerate the rate of displacement of the maxillary incisors or space closure during orthodontic retraction.
- No significant differences were found in tooth inclination, root resorption, or loss of anchorage from the performance of MPOs for the retraction of the maxillary incisors.

## Data Availability

All data generated or analyzed during this study are included in this article. Further enquiries can be directed to the corresponding author.

## Abbreviations

MOPs: Micro-osteoperforations
MOPG: Micro-osteoperforations group
CG: Comparison group
RCCT: Randomized controlled clinical trial
CONSORT: Consolidated Standards of Reporting Trials
CBCT: Cone beam computed tomography
TADs: Temporary anchorage devices
FOV: Field of view
DICOM: Digital Imaging and Communications in Medicine
ANS: Anterior nasal spine
PNS: Posterior nasal spine
ICC: Intraclass correlation coefficient
RAP: Rapid acceleratory phenomenon
